# PROMETHEUS: A Copper-Based Polymetallic Catalyst for Automotive Applications. Part I: Synthesis and Characterization

**DOI:** 10.3390/ma14030622

**Published:** 2021-01-29

**Authors:** Iakovos Yakoumis

**Affiliations:** Monolithos Catalysts & Recycling Limited, 11476 Athens, Greece; yakoumis@monolithos-catalysts.gr; Tel.: +30-210-645-0106

**Keywords:** copper catalyst, platinum group metals, catalytic converter, PGMs

## Abstract

According to the strict European exhaust emissions standards that have been imposed by European legislation there is an elevated need for the decrease of the toxic gas emissions from vehicles. Therefore, car manufacturers have implemented a series of catalytic devices in the aftertreatment of the engine to comply with the standards. All catalytic devices (such as three-way catalysts, diesel particulate filters and diesel oxidation catalysts) accumulate concentrated loading of platinum group metals (PGMs, platinum, palladium, rhodium) as the active catalytic phase. Thus, the demand for PGMs is constantly increasing with a subsequent increase in their market prices. As a result, the research on catalytic converters of high activity and reduced cost/PGM loading is of great interest. In the present work, the Prometheus catalyst, a polymetallic nanosized copper-based catalyst for automotive emission control applications, is presented in two different metal loadings (2 wt% and 5 wt%) and metal ratios (Cu/Pd/Rh = 21/7/1 and Cu/Pd/Rh = 21/7/3). For the first time, a three-metal (copper, palladium, rhodium) nano-catalyst has been synthesized and characterized on a large scale. By using copper as an active catalytic phase, a reduction of PGMs loading is achieved (up to 85%) resulting in a novel catalytic device with similar or improved catalytic performance compared to commercial ones. The Prometheus catalyst is prepared by a wet impregnation method, using as a carrier an inorganic mixed oxide (CeZrO_4_) exhibiting elevated oxygen storage capacity (OSC). The heterogeneous catalytic powders produced were characterized by both spectroscopic and analytical methods. The metal content and ratio were determined by inductively coupled plasma mass spectrometry (ICP-MS), X-ray fluorescence (XRF) and energy-dispersive X-ray spectroscopy (EDS). The morphology and the catalyst particle size were determined with scanning electron microscopy (SEM) and X-ray diffraction (XRD). The investigation revealed homogeneous particle formation and dispersion. The deposition of the metal nanoparticles on the porous inorganic carrier was verified with N_2_ sorption. Catalytic performance and reactivity of a catalyst (pure wash coat) with molar ratio 21/7/1 and a full-scale Prometheus catalyst with the desired loading of 15 g/ft^3^ were tested on an in-house synthetic gas bench (SGB) for the abatement of CO, CH_4_ and NO, both presenting high catalytic activity.

## 1. Introduction

**Figure 1:**
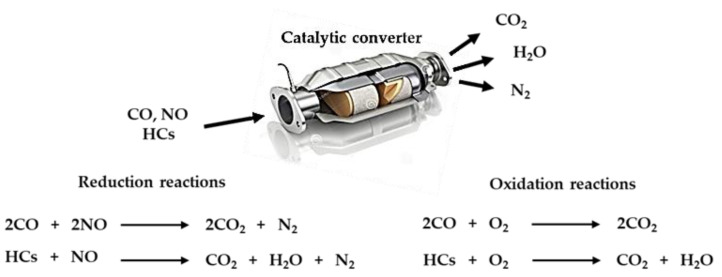
Reactions catalyzed by a catalytic converter.

The growth of the vehicle fleet and the extensive use of cars during the last decades are the main causes of the observed increasing trend of the emissions originated from the transport sector. As a result, strict emissions standards which are presented in Table 1 have been imposed for the reduction of the emissions of toxic gases by the European Commission (EC). Moreover, the need for automotive industries for catalytic converters of high efficiency is essential, since they represent a key part of the exhaust system of every modern vehicle with internal combustion engine. Such catalytic converters, named three-way catalysts, demand higher content of the catalytic phase, which consists mainly of platinum, palladium and rhodium (platinum group metals, PGMs), leading subsequently to increased cost for the manufacturers and the automotive industry. It must be mentioned that palladium and platinum are the active components for CO and HC oxidation and the main role of rhodium is in NOx reduction (Figure 1) [1].

Additionally, PGMs have been characterized as critical raw materials. Such materials’ substitution is difficult, while demand for them is constantly increasing (annual demand of platinum in Europe is 43 tons, worth €1.4 billion) with a subsequent impact on their market prices CRM (Critical Raw Materials), “raw materials for which there are no viable substitutes with current technologies, which most consumer countries are dependent on importing, and whose supply is dominated by one or a few producers”). Therefore, research on the development of new catalytic converters that combine high activity and reduced cost/PGM loading attracts the interest of the automotive industry and scientific community, for both economic and environmental reasons. The aim of this research is the partial substitution of an amount of PGMs (mainly platinum) with abundant non-PGMs (transition metals). Several efforts have been made to use transition metals as substitutes [2] and among them copper seems to be one of the best choices. As a result, herein is introduced a disruptive innovation in automotive catalysts, by substituting up to 85% of the expensive and critical (for the European economy) platinum group metals with copper nanoparticles as the active catalytic material incorporated in automotive catalysts. Copper presents similar and at the same time high catalytic activity to Pt catalysts for the oxidation of CO and HC [3,4,5,6,7,8], but on the other hand for the NO reduction, the activity of Cu-based catalysts is lower than those of PGMs [9,10,11,12,13].

Shore et al. in 2002 presented a bimetallic catalyst comprising platinum (0.25–0.5 wt%) and copper oxide (8 wt%) on cerium oxide powder produced by the impregnation method in two steps. Initially, the cerium oxide powder was impregnated with an aqueous copper nitrate solution and then calcined using a two-step process at 120 °C and at 500 °C for 2 h, respectively. Then, the received powder was impregnated with an aqueous solution of alkali-free-amine-solubilized platinum hydroxide and finally the produced material was calcined again under the same conditions. The bimetallic catalyst was active towards the carbon monoxide remediation, above 90 °C reaction temperatures [14]. Later, a Cu-Ce-O_x_ binary system with various Cu content (from 3 to 20 wt%) has been prepared by a one-pot solvothermal method, characterized and evaluated as catalysts for the low-temperature CO oxidation. As reported these nano porous catalysts containing copper loading 5–7 wt% were very active for the CO oxidation and showed higher catalytic activity with a complete CO conversion at 80 °C than those of 10-Cu-Xe-O_x_ prepared by impregnation using commercial CeO_2_ as support [15]. A series of mesostructured CuCe catalysts were prepared via a Cetyltrimethylammonium Bromide (CTAB)-assisted co-precipitation method and tested for low-temperature CO oxidation. As reported by the authors, Cu could enter the CeO_2_ matrix with a fluorite-like structure to form a solid solution, but a higher Cu content causes the formation of bulk CuO. The synergistic interaction of Cu and Ce species can produce large amounts of oxygen vacancies and improve the redox ability, thereby favoring the catalytic activity. Among the obtained catalysts, Cu_0.1_Ce exhibits the highest catalytic activity for CO oxidation with the lowest T_50_ = 59 °C and high catalytic stability [16]. On the other hand, activity measurements were made of the NO + CO reaction over a series of CuO/CeO_2_ and CuO/γ-Al_2_O_3_ catalysts with various loading amounts of CuO at low-temperatures. According to Lin Dong et al., NO conversion has an intimate relation with the loading amount of CuO and the supports used at temperature lower than 200 °C, and the activities decreased in the order: CuO/CeO_2_ > CuO/γ-Al_2_O_3_ > crystalline CuO. Under these reaction conditions, the activity of the catalysts strongly depended on the surface dispersed copper oxide species. When the temperature was increased to 300 °C, NO completely converted to N_2_ over the catalysts, which meant that the reaction was strongly affected by temperature [17].

Although previous research efforts have been made to replace noble metals with low-cost metals no successful commercial product has been presented so far, resulting in the use of exclusively noble metals for the production of catalytic converters with continuously higher metal loading. Similar catalysts with the herein presented Prometheus catalyst have been published in the literature for the treatment of engine flue gases [18,19,20,21] without being described mixtures that are designed based on the ternary synergy between the copper, the noble metal/metals and the high ionic conductivity or oxygen storage capacity of the ceramic carrier in order to achieve high catalytic activity and selectivity.

**Figure 2:**
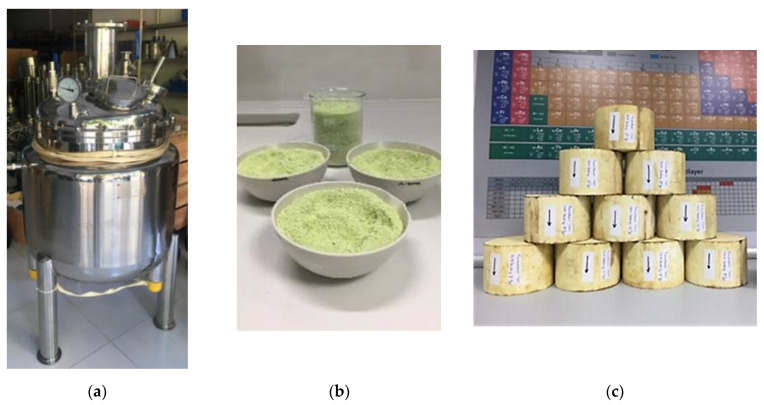
Large scale synthesis of Prometheus monolithic catalysts: (**a**) 150 L jacketed SS316 tank reactor; (**b**) Prometheus catalytic powder; (**c**) Prometheus monolithic catalysts.

In the present work, Prometheus catalyst, a polymetallic nanosized copper-based catalyst for automotive emission control application, is presented. For the first time, a three-metal (copper, palladium, rhodium) nano-catalyst has been synthesized and characterized in lab and subsequently in large scale (Figure 2). The use of copper, an element of higher abundance than platinum in the Earth’s crust, as active catalytic phase affords a final catalytic device with similar or improved catalytic performance and up to 85% less use of PGMs as compared with commercial catalysts.

The Prometheus catalyst is produced by a wet impregnation method, using an inorganic mixed oxide as a carrier (Ce_0.68_Zr_0.32_O_2_) for the dispersion metallic active phase. CeO_2_-ZrO_2_ mixed oxide has been widely used as catalyst support due to its high surface area, thermal stability and oxygen storage capacity (OSC) [22]. The incorporation of zirconium into ceria lattice creates a high concentration of defects, which can stabilize CeO_2_ against sintering, enhance the thermal stability and the efficiency of the TWCs [23]. Several characterization techniques were employed to gain an insight into the physicochemical characteristics of the catalyst, such as inductively coupled plasma mass spectrometry (ICP-MS), X-ray fluorescence (XRF), scanning electron microscopy (SEM), X-ray diffraction (XRD), Raman spectroscopy and N_2_ sorption. The catalytic activity of Prometheus was evaluated in the abatement of CO, CH_4_ and NO.

## 2. Materials and Methods

### 2.1. Prometheus Nano-Catalyst Powder Preparation

**Figure 3:**
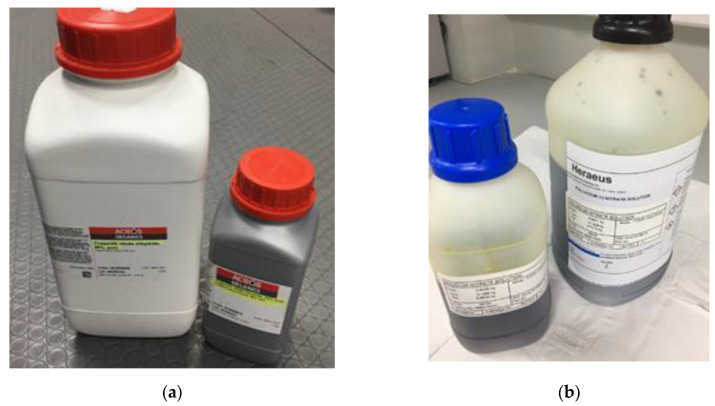
Cu, Pd, Rh metal precursors: (**a**) copper nitrate precursor; (**b**) platinum group metal (PGM, Pd and Rh) nitrate precursor solutions.

**Figure 4:**
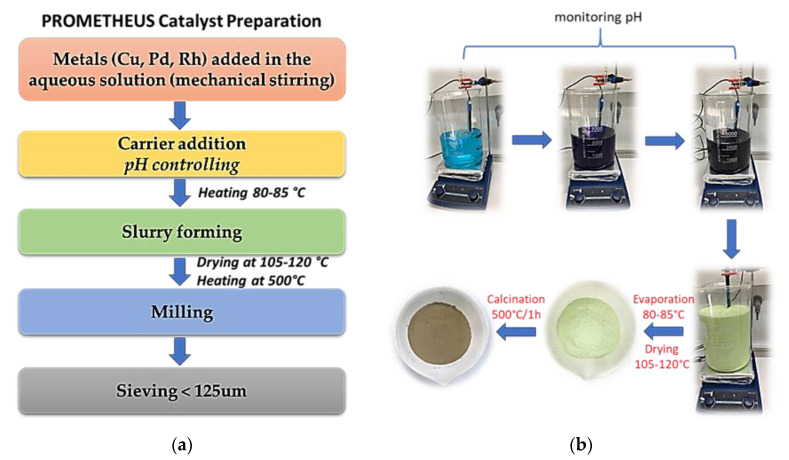
Preparation of Prometheus catalyst: (**a**) catalyst preparation flow chart; (**b**) schematic description.

All chemicals used in this study for the preparation of Prometheus catalytic powder were commercial and were used without further purification. The following chemical reagents were used: ceria-zirconia mixed inorganic oxide, Ce_0.68_Zr_0.32_O_2_ (CZ, Wanfeng Technology, Shaoxing, China) and ammonium hydroxide solution (Merck, percent concentration 25 wt%). As metal precursors were used Cu, Pd, Rh nitrates either in solid or solution form. In more detail, copper (II) nitrate trihydrate (Acros Organic, purity 99%) was added in solid form (Figure 3a). On the other hand, palladium (II) nitrate solution (Heraeus, solution assay 17.94 wt%), and rhodium (III) nitrate solution (Hereaus, solution assay 9.27 wt%) were added in solution form (Figure 3b). The heterogeneous Prometheus catalyst was synthesized by the conventional wet impregnation method according to Figure 4 in two different metal loadings and metal concentrations.

Mass calculations of materials used were performed, in order to achieve the desired metal loading of 2 wt% and 5 wt%, at molar ratio Cu/Pd/Rh = 21/7/1 and Cu/Pd/Rh = 21/7/3, respectively. Metal precursors were firstly dissolved partially in distilled water (DI) and then Ce_0.68_Zr_0.32_O_2_ support (CZ) was added slowly under mechanical stirring. At the same time, the pH was adjusted at pH 11 units with 25% aq. NH_4_OH and then the solution was heated at 80–85 °C under continuous mechanical stirring. The water solvent was evaporated and the catalyst was received in powder form. The catalyst was dried overnight at 105–120 °C and then calcinated at 500 °C for 1 h (heating ramp. 10 °C min^−1^).

Finally, the catalytic powders prepared were sieved at <125 μm in a high frequency sieve shaker (model CZS-1, Nanjing T-Bota Scietech Instruments and Equipment Co., Ltd., Nanjing, China), obtaining a fine granulometry, in order to improve the specific surface area required for further processing. Regarding the impregnated 15 g/ft^3^ trimetallic cordierite 21/7/1 2%, the monolith was grinded to pieces and then subjected to milling and sieved in order to obtain particle size < 125 μm. The obtained powder was homogenized and a sample was obtained for the catalytic performance measurements. Prometheus catalyst produced with metal loading 2 wt% and metal concentration Cu/Pd/Rh = 21/7/1 will be referred to as PROM2 and Prometheus catalyst produced with metal loading 5 wt% and metal concentration Cu/Pd/Rh = 21/7/3 will be referred to as PROM5. Monometallic copper-based catalyst, namely CuCZ with metal loading 2 wt% was also prepared under the same experimental conditions and procedure in order for the single metal catalyst performance to be studied.

### 2.2. Prometheus Monolithic Catalysts

**Figure 5:**
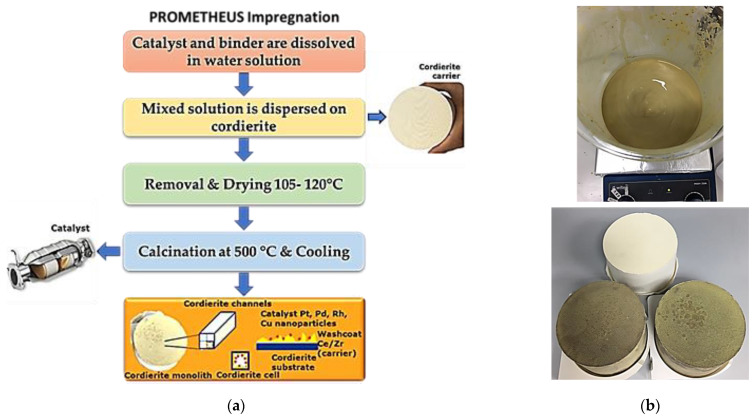
(**a**) Flow chart Prometheus impregnation; (**b**) aqueous slurry preparation and ceramic monolith before and after the impregnation into the aqueous slurry.

Prometheus catalytic powder was impregnated on a ceramic cordierite-based monolith, (Mg, Fe)_2_Al_4_Si_5_O_18_) in order to simulate the conditions of the catalyst performance while installed on a vehicle. Impregnation steps are described in Figure 5.

Prior to the impregnation, the ceramic monolith bulk characteristics of the monolith were determined (dimensions, volume, bulk density: g/ft^3^) and dried at 90 °C (1 ft = 30.48 cm). In order for the desired loading to be obtained for ceramic monolith (15 g PGMs/ft^3^, PGMs: Pd and Rh), the required mass of the catalytic powders PROM2 was calculated, respectively. An aqueous slurry containing the appropriate amount of catalytic powder and γ-Al_2_O_3_ boehmite (Disperal P2, Sasol) as binder, was prepared under stirring at room temperature while the pH value was adjusted to pH 7 with 25% aq. NH_4_OH buffer solution. The ceramic monolith was dipped into the slurry, remained submerged for a few minutes and then, removed while shaking for excessive slurry removal from the channels. Then, the monolith was left to dry at 90 °C for 1 h, while rotating continuously around its axis, in order to avoid capillary forces phenomena in the cells of the cordierite. The impregnated monolith was left overnight at 90 °C and after that the impregnation procedure was repeated according to the above conditions. The impregnated monolith was finally calcinated at 500 °C for 1 h (heating ramp. 10 °C min^−1^) and then blown with air in order to remove the non-coated particles. The metal loading of the monolith was determined by the weight of the monolith before and after the impregnation.

### 2.3. Materials’ Characterization

PROMETHUS catalyst was subjected to physicochemical characterization in order for physical properties and morphology to be determined. Crystallinity of the samples and type of crystalline phases present in the materials were identified with powder X-ray diffraction (XRD), scanning electron microscopy (SEM) and Raman spectroscopy. The metal loading of Prometheus catalyst was determined with ICP-MS, XRF, and EDS. Finally, N_2_ physisorption isotherm curves were obtained to investigate the Brunauer–Emmett–Teller (BET) surface area and Barrett–Joyner–Halenda (BJH) pore size distribution of the prepared samples.

The X-ray diffraction measurements were obtained with a Rigaku X-ray diffractometer (XRD) (Rigaku Corporation, Tokyo, Japan) with a Bragg–Brentano geometry using Cu Ka radiation (λ = 1.54178 Å) and a graphite monochromator in the diffracted beam. The microstructural parameters were evaluated by fitting the full XRD patterns using MAUD (materials analysis using diffraction), a diffraction/reflectivity analysis program based on the Rietveld method. The morphology and metals distribution on the substrate surface of the catalytic powder was determined via SEM/EDS, using a QUANTA FEI SEM and EDAX detector (FEI Company, Hillsboro, OR, USA). Further analysis of the catalyst morphology has been performed via Image J program. All Raman spectra were collected using a 532 nm Raman Spectrometer. The spectral acquisition consisted of 50 accumulations of 2 s for each sample under a 100× objective lens.

Two analytical methods were applied for the evaluation of the elemental content of the catalytic powders: ICP-MS and XRF. In case of ICP-MS each sample was weighted (0.15 g) directly in PTFE.PFA digestion vessels in duplicate and 8 mL of aqua regia (6 mL HCL and 2 mL HNO_3_) was added to each vessel. Samples were digested in closed high pressure microwave-assisted oven, filtered before analysis and diluted to 15 mL with deionized water. Concentration of copper, palladium and rhodium was determined by using a standard Agilent 8800 Triple Quadrupole ICP-MS. The standard configuration is composed of an x-lens, a Peltier-cooled double pass Scott-type spray chamber, a glass concentric nebulizer, and a one-piece quartz torch with internal injector. The measuring conditions of the spectrometer were as follows: RF generator power: 1550 W; plasma gas flowrate: 15 L/min; auxiliary gas flowrate: 0.90 L/min and nebulizer gas flowrate: 1.09 L/min. XRF analysis took place for the catalysts using the equipment Vanta Olympus (2017, Waltham, MA, USA). The samples were prepared in the form of pressed powder in polypropylene cups (m = 5 g). Catalysts were ground in a blade mill, where particle size was reduced (<125 μm) and then dried at 120 °C for 2 h before sample preparation and XRF analysis. Each sample was measured with 10 repeated scans, 90 s each. In order to obtain high accuracy measurements of the metal loading XRF instrument was calibrated for each metal additionally to the internal calibration of the instrument. Pd was calibrated in the loading range of 800–1000 ppm, Rh in the loading range of 230–330 ppm, and Cu in the loading range of 0.7–12%.

The surface area and pore size distribution of the catalysts were determined by N_2_ sorption isotherms at liquid N_2_ (−196 °C) on a Sorptomatic 1990 instrument (Thermo Scientific, Waltham, MA, USA). Specific surface area and pore size distribution were calculated by BET theory and the BJH method, respectively. Typically, approximately 1.5 g of each sample was loaded into the BET tube and degassed at 300 °C for 19 h under high vacuum prior analysis in order to completely remove the chemisorbed water from sample surface.

### 2.4. Catalytic Activity Measurement

**Figure 6:**
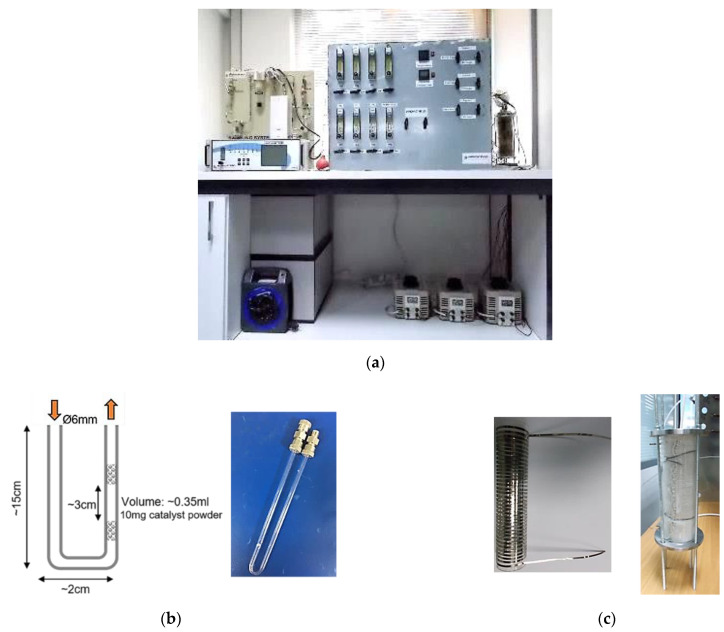
(**a**) Synthetic gas bench (SGB); (**b**) U-shaped quartz reactor for activity testing of catalyst powders; (**c**) tube experimental electrical furnace.

The catalytic activity of the following materials, copper monometallic catalytic powder, Prometheus trimetallic catalytic powder (PROM2) and the full-scale 15 g/ft^3^ Prometheus monolithic trimetallic catalyst was tested on an in-house synthetic gas bench (SGB, Figure 6a) towards the oxidation of CO and CH_4_ and the reduction of NO. The powder catalysts were sieved (<125 μm) and loaded into a U-shaped reactor (10 mg, 0.35 mL) as shown in Figure 6b and then inserted in a glass tube electrical furnace, where temperature was controlled and monitored with a k-type thermocouple (Figure 6c).

The gas experimental conditions were designed under simulated gasoline engine exhaust conditions. Therefore, catalytic activity tests were both performed under reducing and oxidizing conditions, e.g., λ ≈ 0.99 and λ ≈ 1.03 (lambda sensor λ = A/F; A: air, F: Fuel), in the temperature range between 100–400 °C. The concentration of the synthetic gas which was used to simulate the vehicle exhaust conditions is presented in Table 2. Water was also added to the feed by passing nitrogen through a saturator with deionized water at 65 °C.

The gas feed (CH_4_, CO, O_2_, NO_x_) to the reactor and the converted gases (CO_2_, N_2_) were determined with an online multi gas analyzer (GA-200, HNL) and the conversion rate was measured according to the Equation (1). Gas analysis was performed using NO_2_, CO, NO and O_2_ electrochemical detectors, CH_4_ catalytic pellister and a non-dispersive infrared CO_2_ detector.

(1) $$Xi=\;\frac{C_{in}-C_{out}}{C_{in}}\;\times \;100\%$$

where *Xi* refers to gas conversion (%), *C_in_* to inlet gas (%) and *C_out_* to outlet gas (%).

## 3. Results

### 3.1. Morphology and Structure of the Catalyst

#### 3.1.1. X-ray Diffraction Characterization

**Figure 7:**
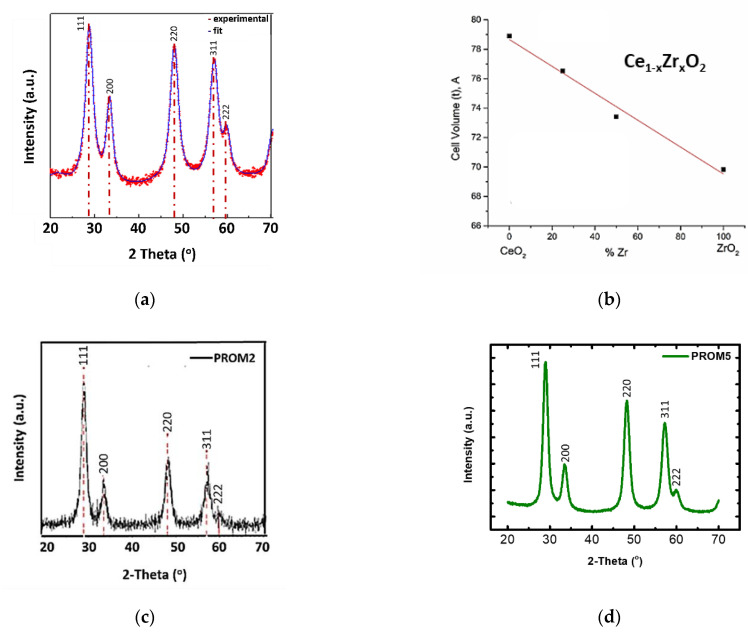
(**a**) XRD pattern (red dots) and fitting (blue line) of the Ce_0.68_Zr_0.32_O_2_ (CZ) sample; (**b**) cubic crystal cell volume of various Ce-Zr solid solutions; (**c**) X-ray diffraction (XRD) pattern of PROM2; (**d**) XRD pattern of PROM5.

The crystal structure of the commercial pure ceramic support material Ce_0.68_Zr_0.32_O_2_ (CZ) was defined by X-ray diffraction (Figure 5a). The experimental pattern has been interpolated using Ce_0.5_Zr_0.5_O_2_ tetragonal phase with starting parameters of a = b = 3.7205 Å and c = 5.3039 Å. The fit profile (blue line, Figure 7a), carried out by MAUD program satisfactory interpolate the experimental data (Rwp (%) = 2.8). The structural and microstructural data were determined by Rietveld refinement according to which crystallite size was 91 Å (a: 3.773 Å, c: 5.343 Å) and crystallite volume 76.06 Å^3^. In addition, the cell volume of different Ce_x_Zr_1−x_O_2_ solid solutions is presented in Figure 7b, using reported values in the literature for crystallite cell volume at various solid solution composition. Our aim was to estimate (confirm) the atomic composition of supplied CZ solid solution. Given the measured XRD analysis cell volume of ~76 Å^3^, what can be estimated is that the oxide support has an atomic composition of about Ce_0.70_Zr_0.30_O_2_. However, in order to verify the crystal structure of CZ (cubic or tetragonal structure), Raman spectra was also collected in the 1500–50 cm^−1^ spectral region [24,25]. Furthermore, according to the XRD pattern (Figure 5a), the main reflection bands appear at 28.8, 33.5, 48, 57.2, 59.8 2θ angles. These bands are attributed to [111], [200], [220], [311], and [222] crystal lattice planes, respectively lattice planes of cubic fluorite CeO_2_ [26]. The 2θ angle position and the relative intensities of the peaks indicate that CZ is developed on cubic crystal structure. These bands appear also in XRD patterns of the catalysts PROM2 and PROM5 (Figure 7c,d), indicating the appearance of the same crystal phases in both samples. However, the bands of 48° and 57.2° in the sample PROM2 decreases in relative intensities with respect to the main band of 28.8°, indicating that [220] and [311] crystal lattices decrease upon catalytic nanoparticle impregnation. On the other hand, in the PROM5 XRD pattern the corresponding bands recover their relative intensity with respect to the main band 28.8°, indicating that these lattices become visible again. Moreover, the metal atoms are not forming XRD-detectable particles on the support (CZ) possible due to the low loading or/and high dispersion [27,28].

#### 3.1.2. Raman Analysis

**Figure 8:**
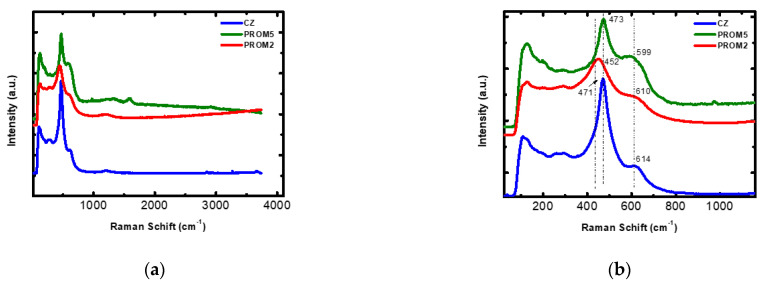
Raman spectrum of the samples CZ, PROM2, PROM5 (**a**) 10–4000 cm^−1^; (**b**) 10–1100 cm^−1^.

Collected spectra of the support material CZ and Prometheus catalysts PROM2 and PROM5 are presented in Figure 6. Most pronounced spectral changes are observed in the spectral range of 1500–50 cm^−1^. The CZ substrate spectra (Figure 8a) has been compared to Raman spectra and phase diagrams of the solid solution of Ce_x_Zr_1−x_O_2_ from literature, where only two tetragonal phases, namely t’ and t” are present. [29]. Furthermore, according to our spectra the strong band at 471 cm^−1^ is attributed to O-Ce-O stretching vibrational mode in the cubic fluorite phase (F2 g). This band exhibits red shift at 448 cm^−1^ for PROM2 catalyst (Figure 8b) which might be attributed to the distortion of the Ce-O vibrational mode and structure as well, due to the substitution of some Ce^4+^ ions with Cu^2+^, Pd^2+^, Rh^3+^ [30]. Moreover, this band is blue shifted at 473 cm^−1^ for PROM5 (Figure 8b) indicating the presence of the nano catalyst on the structure of the substrate, where the lattice might have recovered the same vibrational modes and lattice energy as it had without the catalyst phase, indicating a possible doping of the substrate with the metals Cu, Pd and Rh [31,32].

Finally, the bands at 1205, 1207, 1225 cm^−1^ for CZ, PROM2 and PROM5 respectively, and the low frequency bands at 614, 610 and 599 cm^−1^ for CZ, PROM2 and PROM5, as well, correspond to the defect-induced bands Ce-O of the cubic phase of the CZ substrate. These spectral changes, are in accordance to XRD results, where [220] and [311] crystal planes are affected by the addition of the catalytic phase on the substrate, indicating that the possible doping and Ce ion substitution might have taken place upon substrate impregnation.

#### 3.1.3. Scanning Electron Microscopy (SEM)—Energy-Dispersive X-ray Spectroscopy (EDS) Analysis

**Figure 9:**
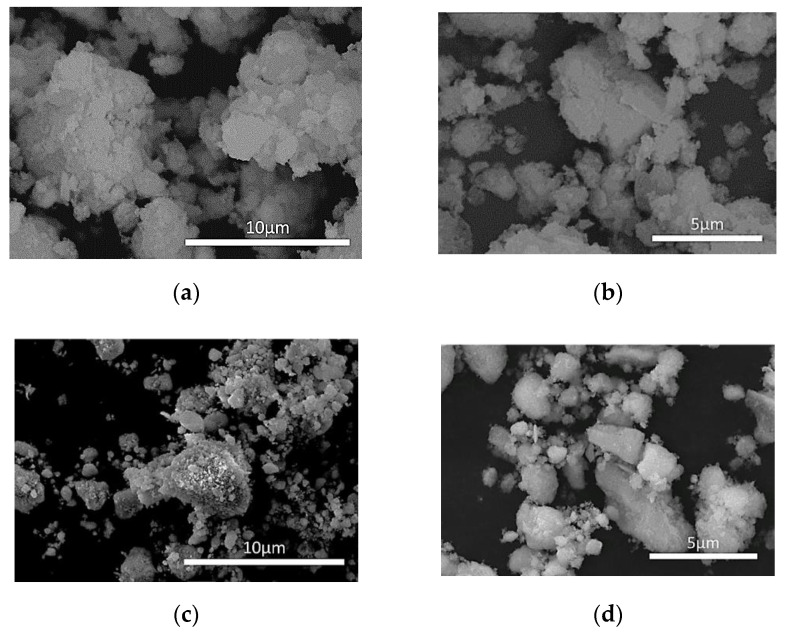
Scanning electron microscope (SEM) images of catalyst PROM2 (**a**) ×5000; (**b**) ×7000 and catalyst PROM5 (**c**) ×1000; (**d**) ×3000.

The morphology of the Prometheus catalytic powders, PROM2 and PROM5 was determined via SEM/EDS, using a QUANTA FEI SEM and EDAX detector (Figure 9). Further analysis of the catalyst morphology was performed via the Image J program according to which the mean particle size for PROM2 and PROM5 catalysts was calculated at 1.42 μm (×5000) and 2.46 μm (×3000), respectively. The surface morphology of trimetallic catalysts seems rough (Figure 9a,d), revealing agglomerated particulates.

**Figure 10:**
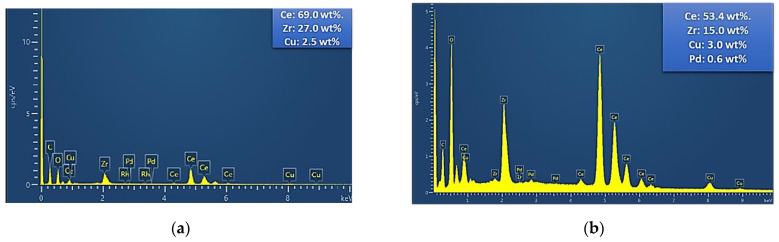
Mapping analysis of the catalysts: (**a**) PROM2; (**b**) PROM5.

**Figure 11:**
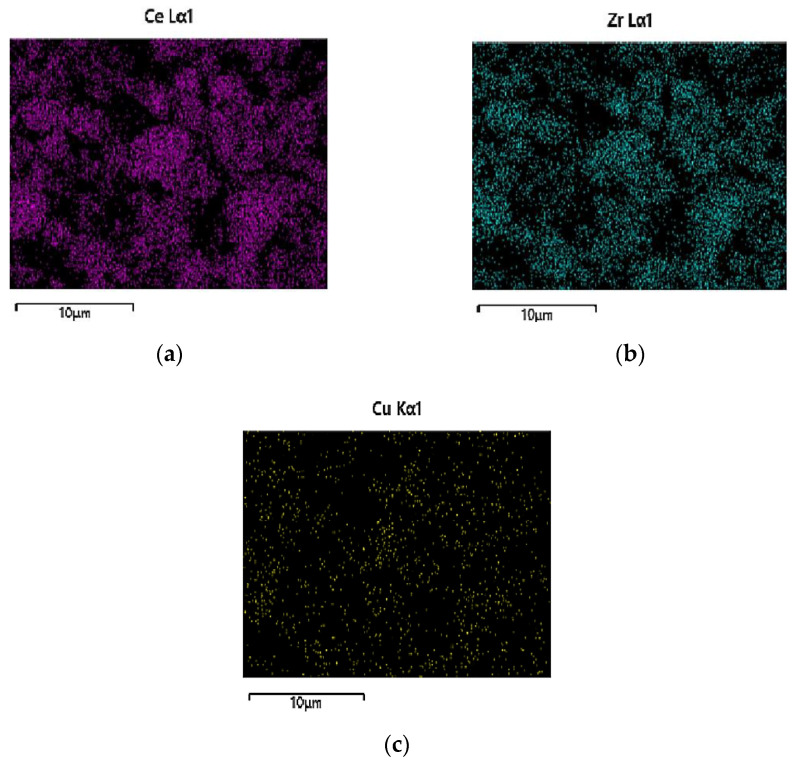
Distribution of: (**a**) Ce; (**b**) Zr; (**c**) Cu for PROM2 catalyst.

**Figure 12:**
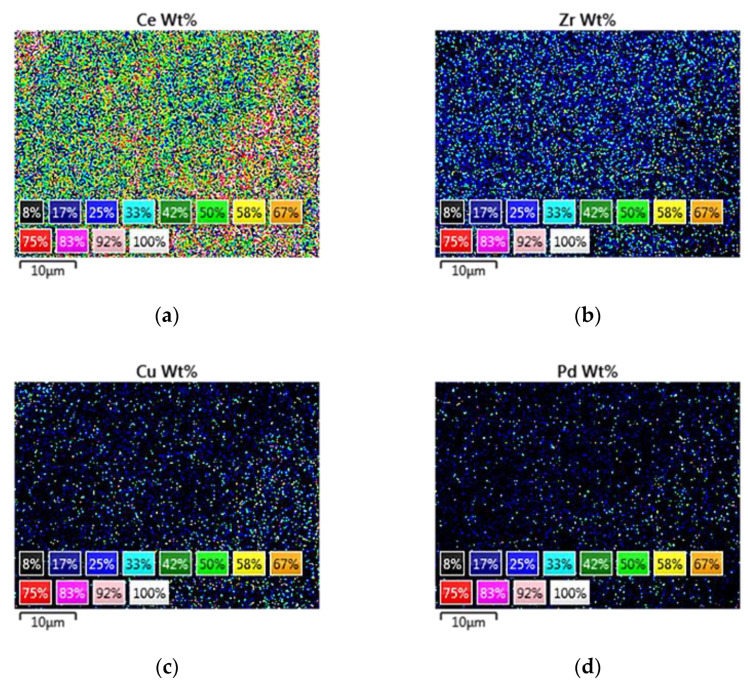
Distribution of: (**a**) Ce; (b) Zr; (**c**) Cu; (**d**) Pd for PROM5 catalyst.

Moreover, mapping analysis over catalysts PROM2 and PROM5 (Figure 10) revealed a homogenized grain distribution for Ce (L), Zr (L), Cu (K), Pd (L) (Figure 11 and Figure 12). In the case of PROM2, the elements Pd and Rh were not detectable, due to the low detection limit of the instrument, while in the case of PROM5 the detection of Rh was also impossibly probable due to low content (<1 wt%). According to the quantitative analysis results (Table 3), Ce content is higher than Zr, as expected from Ce_0.68_Zr_0.32_O_2_ stoichiometry (Ce 75%, Zr 25%) and Cu is the dominant metal, as expected from Cu/Pd/Rh = 21/7/1 and Cu/Pd/Rh = 21/7/3 mass ratio.

#### 3.1.4. N_2_ Physisorption

**Figure 13:**
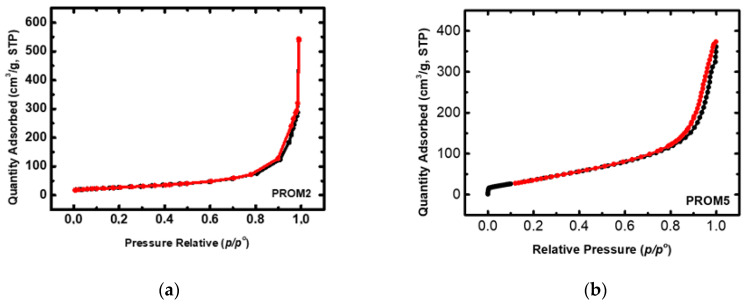
Isotherm curve of N_2_ physisorption for Prometheus tri-metallic catalysts: (**a**) PROM2; (**b**) PROM5.

The specific area of the trimetallic Prometheus catalyst samples PROM2 and PROM5 was determined by the N_2_ sorption method (Figure 13). Outgassing of the samples was conducted at 300 °C in order for the humidity to be removed and the temperature rate was controlled at 1 °C/min with an initial temperature set at 30 °C. BET surface area of the PROM2 catalyst was slightly decreased (97 m^2^/g) compared to commercial CZ (108 m^2^/g) probably due to the immobilization of metal nanoparticles on the surface of the support and its pore blockage. In addition, PROM2 catalyst presents a mesoporous and macroporous structure with maxima pore sizes (BJH method) 11.9 nm and 148 nm. On the other hand, BET surface area of PROM5 catalyst was increased (148 m^2^/g) compared to CZ, presenting a mesoporous structure with maxima pore sizes (BJH method) 21 nm and 35 nm. This may be due to the dispersion state of metal nanoparticles being quite high and there is a better organization of the structure.

### 3.2. Catalyst Loading

#### 3.2.1. Inductively Coupled Plasma Mass Spectrometry (ICP-MS), X-ray Fluorescence (XRF) Analysis

**Figure 14:**
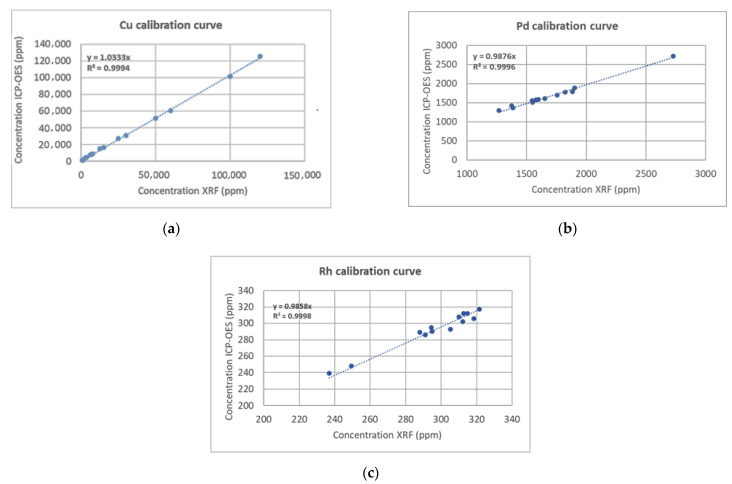
Calibration curves for: (**a**) Cu in the loading range 0.7–12%; (**b**) Pd in the loading range < 1270–2730 ppm; (**c**) Rh in the loading range 230–330 ppm.

The elemental content of PROM2 and PROM5 was evaluated by ICP-MS and XRF analysis, with respect to EDS analysis (Table 4). In order for more accurate measurements to be obtained the XRF instrument was calibrated for each metal separately, additionally to the internal calibration of the instrument. In the case of precious metals (Pd and Rh) 13 spent automotive catalyst samples with varied metal concentration were used for calibration. On the other hand, in the case of copper, commercial samples with known metal concentrations were used for calibration (OREAS CRMs, ORE Research & Exploration Pty Ltd., Bayswater North, VIC, Australia). The nominal concentration value was already measured by the ICP-MS method. The calibration curves obtained by the aforementioned samples for Pd, Rh and Cu are presented in Figure 14. The slope for each curve was calculated with x-axes average concentration from 10 scans for each sample and y-axes nominal concentration measured by ICPMS (Inductively Coupled Plasma Mass Spectrometry). Pd has been calibrated in the loading range of <1270–2730 ppm, Rh in the loading range of 230–330 ppm and Cu in the loading range of 0.7–12%. Comparison of the results for copper and precious metals obtained by XRF and ICP-MS techniques clearly show that they exhibit standard deviation lower than 5% confirming the presence of metal nanoparticles in the catalysts PROM2 and PROM5, respectively.

#### 3.2.2. Morphology of Full-Scale Prometheus Catalyst

**Figure 15:**
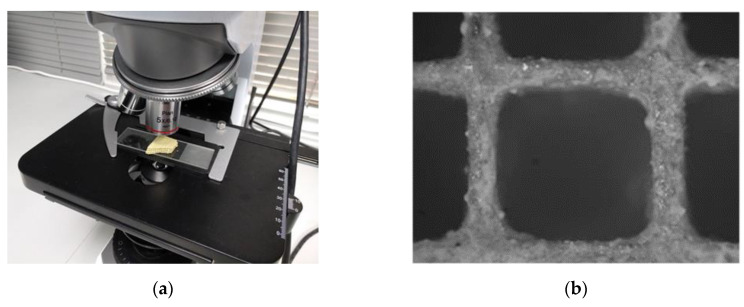
(**a**) Optical microscope used to estimate the thickness and morphology of the washcoat in TWCs; (**b**) optical microscopy cross-section images of the 15 g/ft^3^ Prometheus monolithic catalyst.

The morphology of a monolithic catalyst was examined with optical microscopy, using a microscope purchased by AmScope (ME520 series), equipped with a microscope digital camera 14 MP ultrafine color (Figure 15). Small pieces of the sample monoliths were placed under the objective lenses and could be moved in the vertical direction to focus and obtain a resolution suitable for measuring the thickness of the wash coat. Objective lenses of 5×, 10×, 20× and 100× magnification were used. As shown, only one coating layer can be distinguished on the walls of the cordierite matrix; corresponding to the catalyst, having a thickness of approximately 50–150 μm.

### 3.3. Catalytic Performance

The following prepared catalysts were experimentally tested regarding their catalytic activity for the abatement of 3 toxic gases CO, CH_4_ and NO using the in-house synthetic gas bench set up under rich and lean-burn conditions (λ = 0.99 and λ = 1.03), respectively. The copper monometallic catalyst powder comprising 2 wt% Cu nanoparticles and Prometheus trimetallic catalyst powder comprising 2 wt% Cu, Pd and Rh nanoparticles (PROM2) are supported over Ce_0.68_Zr_0.32_O_2−δ_ ceramic carrier, respectively. In addition, Prometheus monolithic catalyst powder was evaluated with the desired loading 15 g PGMs/ft^3^ (PGMs: Pd and Rh).

#### 3.3.1. Copper Monometallic and Prometheus Trimetallic Catalytic powders (WashCoats)

**Figure 16:**
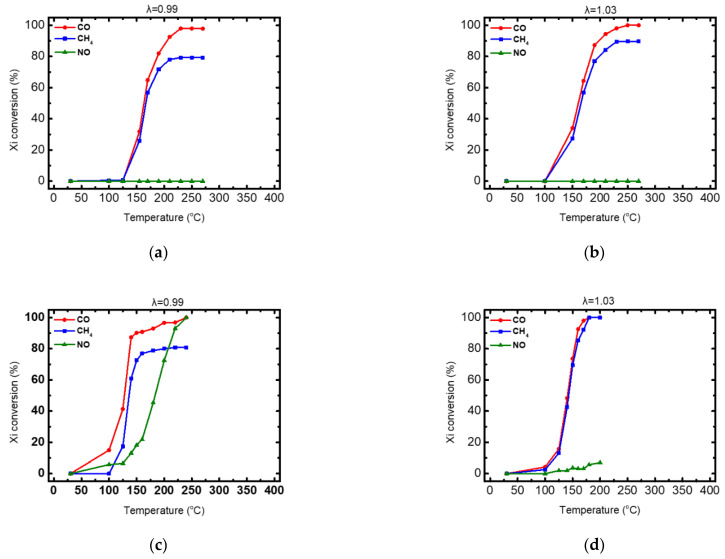
Light-off curves for the conversion of CO, CH_4_ and NO: (**a**) copper monometallic catalyst powder under rich-burn conditions (λ = 0.99); (**b**) copper monometallic catalyst powder under lean-burn conditions (λ = 1.03); (**c**) PROM2 trimetallic catalyst powder under rich-burn conditions (λ = 0.99); (**d**) PROM2 trimetallic catalyst powder under lean-burn conditions (λ = 1.03).

Catalytic activity of monometallic copper catalyst powder and PROM2 trimetallic catalyst powder and their corresponding light-off curves for rich-burn (λ = 0.99) and lean-burn (λ = 1.03) conditions, are presented in Figure 16. Furthermore, important activity indication values for the catalysts’ characterization are summarized in Table 5 for rich-burn (λ = 0.99) and in Table 6 for lean-burn (λ = 1.03) conditions, respectively.

According to Figure 13 both catalytic powders present significant catalytic activity regarding the oxidation of CO. The CH_4_ oxidation efficiency was high under lean-burn conditions (λ = 1.03) for both catalysts and limited under rich-burn conditions (λ = 0.99). On the other hand, NO reduction was observed only by PROM2 catalyst under rich-burn conditions (λ = 0.99), with copper monometallic catalyst being inactive. In particular, the produced monometallic copper catalyst (pure wash-coat) comprising 2 wt% Cu loading, is active at T > 130 °C under both rich- and lean-burn conditions. CO oxidation efficiency reached ~100% in both condition cases, while CH_4_ efficiency reached 90% under lean-burn, but it was limited to ~79% at rich-burn conditions. No NO reduction reaction was observed either under rich-burn, or under lean-burn conditions, revealing a Cu contribution only to the oxidation reactions. PROM2 trimetallic catalyst (pure wash-coat), comprising 0.552 wt% PGMs loading and 1.448 wt% Cu loading is active at T > 120 °C, under both rich- and lean-burn conditions. CO oxidation efficiency reached ~100% in both condition cases, while CH_4_ efficiency reached 100% under lean-burn but it was limited to ~81% at rich-burn conditions. Finally, NO reduction under rich-burn conditions reached 99% at ~215 °C and total efficiency 100% at 240 °C but on the other hand, only 6% NO reduction efficiency was observed under lean-burn conditions at 200 °C. This is possibly due to surface oxidation of the Rh nanoparticles present in the catalyst.

#### 3.3.2. The 15 g/ft^3^ Trimetallic Prometheus Catalyst Powder

**Figure 17:**
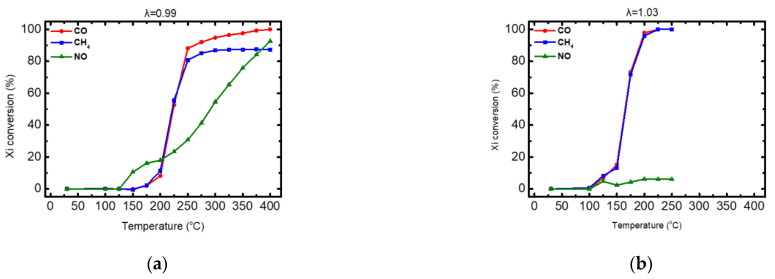
Light-off curves for the conversion of CO, CH_4_ and NO. (**a**) λ = 0.99, (**b**) λ = 1.03.

The produced fresh trimetallic Prometheus monolithic catalyst powder, resulting in 15 g/ft^3^ of PGM (Pd and Rh) and 39.4 g/ft^3^ of Cu, was tested under both rich-burn (λ = 0.99) and lean-burn (λ = 1.03) conditions for the abatement of CO, CH_4_ and NO. Corresponding light-off curves are shown in Figure 17 for λ = 0.99 and for λ = 1.03. Important activity indication values for catalyst characterization are summarized in Table 7.

As shown in Table 6, the Prometheus monolithic catalyst having 15 g/ft^3^ of PGM, is active at T > 190 °C under rich-burn conditions and at T > 150 °C under lean-burn conditions, exhibiting a shift to higher temperatures compared to PROM2 catalyst (pure washcoat) that was active at T > 120 °C in all cases. Namely, CO oxidation efficiency reached ~100% in both condition cases, while CH_4_ efficiency reached 100% under lean-burn but it was limited to ~87% at rich-burn conditions. It is worth noting that the recorded reduction in NO reduction activity (~100 °C temperature shift) of Prometheus monolithic catalyst, compared to PROM2 catalyst (pure wash-coat), could be attributed to the Rh content reduction in the catalyst. On the other hand, poor NO reduction reaction efficiency (~6%) was observed under lean-burn conditions, possibly due to surface oxidation of the Rh nanoparticles present in the catalyst.

It is worth mentioning that the commercial Euro 6d three-way catalytic converters (incorporating loadings in the area of 60–100 g/ft^3^ of PGMs) present similar or lower catalytic activity to the Prometheus catalyst (incorporating loading of 15 g/ft^3^ of PGMs) resulting in a substitution of PGMs between 75–85% for all cases.

## 4. Conclusions

A novel trimetallic copper-based nano-catalyst comprising copper, palladium and rhodium supported on Ce_0.68_Zr_0.32_O_2_ carrier (CZ) was produced and physiochemically characterized. The catalytic powder was successfully deposited on cordierite supports affording catalysts with loading of 15 g PGMs/ft^3^. The morphology and the structure of the catalytic powder was examined, revealing a homogeneous dispersion of the metal particles on the carrier’s surface (CZ, Ce_0.68_Zr_0.32_O_2_). The metal content was determined with analytic techniques (ICP-MS and XRF). Furthermore, the catalytic activity of the fresh monometallic copper catalyst and the trimetallic catalyst (15 g PGMs/ft^3^) were examined in a synthetic gas bench (SGB). The experimental results revealed an active catalyst for the abatement of CO, NO and CH_4_ and, finally, we may propose the following mechanism according to which the high activity and stability of the catalyst is mainly attributed to the existence of Cu(I) species in the system and the destabilization of Cu (II) species, both due to e-transfer effects from the Rh, Pd metals to copper and strong metal–support interaction phenomena. Oxygen ion species vacancies on the wash-coat support are favored, which results in weakening of the interaction between the adsorbed electronegative species from the gas phase to the surface metal oxide species. In addition, the ability of Ce(III) to be oxidized by steam in the temperature range 300–500 °C and produce H_2_(g) which can then cause the reduction of Cu(I) to Cu, plays an important role. The latter step is of particular importance since the metal Cu formed therein serves the reduction of Ce(IV), thus completing the Ce(III)/Ce(IV) oxidation cycle. In the aforementioned synergy, it is also worth mentioning the steam chemisorption recorded on the surface of CuPt bimetallic catalysts, thus contributing to the critical step of Ce(III) oxidation and the formation of H_2_. For this mechanism to be established and confirmed, XPS measurements should be obtained, and this study should be included in our future work [33,34,35].

## 5. Patents

European Patent of Prometheus: copper and noble metal polymetallic catalysts for engine flue gas treatment. EP3569309. Applicant Monolithos Catalysts and Recycling Ltd., Inventor: Iakovos Yakoumis.

## Figures and Tables

**Table 1:** European Union (EU) emissions standards for passenger cars (Category M1 ^1^).

Stage	Date	CO (g/Km)	ΤHC (g/Km)	HC + NOx (g/Km)	NOx (g/Km)	PM (g/Km)	PN/Km
Euro 1	1992	2.72	-	0.97	-	-	-
Euro 2	1996	2.2	-	0.5	-	-	-
Euro 3	2000	2.3	0.2	-	0.15	-	-
Euro 4	2005	1.0	0.1	-	0.08	-	-
Euro 5	2009	1.0	0.1	-	0.06	0.005	-
Euro 6	2014	1.0	0.1	-	0.06	0.005	6.0 × 10^11^

^1^ At the Euro 1.4 stages, passenger vehicles >2.500 kg were type approved as Category N1 vehicles.

**Table 2:** Selected gas mixtures concentrations for λ ≈ 0.99 and λ ≈ 1.03.

Gas Component	Rich-Burn Conditions λ ≈ 0.99	Lean-Burn Conditions λ ≈ 1.03
CO	1%	1%
CO_2_	12%	12%
O_2_	0.91%	0.95%
NO	800 ppm	800 ppm
CH_4_	2500 ppm	2500 ppm
H_2_O	10%	10%
Pure N_2_	balance	balance
Total vol. Flow rate	300 sccm ^1^	300 sccm ^1^
GHSV ^2^	50.000 h^−1^	50.000 h^−1^

^1^ sccm: Standard cubic centimeters, equal to cm^3^/min. ^2^ GHSV: Gas Hour Space Velocity.

**Table 3:** Energy-dispersive X-ray spectroscopy (EDS) quantification analysis of PROM2 and PROM5.

Catalyst	Element (wt%)				
	Ce	Zr	Cu	Pd	Rh
PROM2	69.0	27.0	2.5	-	-
PROM5	53.4	15.0	3.0	0.6	-

**Table 4:** Comparison of inductively coupled plasma mass spectrometry (ICP-MS), X-ray fluorescence (XRF), techniques for catalyst samples PROM2 and PROM5.

Sample	Technique	Elements (wt%)		
		Cu	Pd	Rh
PROM2	ICP-MS	1.47	0.504	0.097
	XRF	1.51	0.494	0.094
PROM5	ICP-MS	2.53	0.823	0.335
	XRF	2.65	1.124	0.481

**Table 5:** Comparison of tested catalysts for the abatement of CO, CH_4_, NO at rich-burn conditions (λ = 0.99).

Catalyst	CO Oxidation (%)				CH_4_ Oxidation (%)				NO Reduction (%)			
	T_50_	T_90_	T_99_	Max. (%)	T_50_	T_90_	T_99_	Max. (%)	T_50_	T_90_	T_99_	Max. (%)
CuCZ	160	200	-	98	160	-	-	79	-	-	-	0
PROM2	130	150	200	100	135	-	-	81	185	215	230	100

**Table 6:** Comparison of tested catalysts for the abatement of CO, CH_4_, NO at lean-burn conditions (λ = 1.03).

Catalyst	CO Oxidation (%)				CH_4_ Oxidation (%)				NO Reduction (%)			
	T_50_	T_90_	T_99_	Max. (%)	T_50_	T_90_	T_99_	Max. (%)	T_50_	T_90_	T_99_	Max. (%)
CuCZ	160	200	240-	100	160	235	-	90	-	-	-	0
PROM2	140	155	175	100	143	165	180	100	-	-	-	6

**Table 7:** T_50_, T_90_, T_99_ and total efficiency of Prometheus monolithic catalyst for the abatement of the CO, CH_4_, NO at rich-burn (λ = 0.99) and lean-burn conditions (λ = 1.03).

	CO Oxidation (%)		CH_4_ Oxidation (%)		NO Reduction (%)	
	λ 0.99	λ 1.03	λ 0.99	λ 1.03	λ 0.99	λ 1.03
T_50_	220	170	220	170	285	-
T_90_	260	190	-	190	390	-
T_99_	375	215	-	215	-	-
efficiency	100%	100%	87%	100%	96%	6%

## Data Availability

The data presented in this study are available on request from the corresponding author.
